# The Influence of Reflowing Process on Electrodeposited Sn-Cu-Ni Lead-Free Solder Alloy

**DOI:** 10.3390/ma17051034

**Published:** 2024-02-23

**Authors:** Sabrina Patricia State (Rosoiu), Stefania Costovici, Marius Enachescu, Teodor Visan, Liana Anicai

**Affiliations:** 1 Faculty of Medical Engineering, National University for Science and Technology Politehnica Bucharest, 1-7 Gheorghe Polizu Street, 011061 Bucharest, Romania; sabrina.rosoiu@upb.ro; 2 National Institute for Research and Development in Microtechnologies—IMT Bucharest, 126A Erou Iancu Nicolae, 077190 Bucharest, Romania; 3 Center of Surface Science and Nanotechnology, National University for Science and Technology Politehnica Bucharest, Splaiul Independentei 313, 060042 Bucharest, Romania; marius.enachescu@cssnt-upb.ro (M.E.); liana.anicai@cssnt-upb.ro (L.A.)

**Keywords:** Sn-Cu-Ni ternary alloy, electrodeposition, reflowing process, anti-corrosive properties

## Abstract

Sn-Cu-Ni lead-free solder alloy electrodeposited on copper substrate from a deep eutectic solvent (DES)-based electrolyte under direct current (DC) and pulsed current (PC) was subjected to a reflowing process at the industrial company MIBATRON S.R.L. (Otopeni, Romania). The alteration of the alloy’s composition and anti-corrosive properties upon exposure to the reflow process were investigated via Scanning Electron Microscopy (SEM-EDX), X-ray diffraction (XRD), linear sweep voltammetry (LSV) and electrochemical impedance spectroscopy (EIS). Corrosion studies conducted in sodium chloride solution revealed that the system obtained under the DC plating mode (Sn-Cu-Ni-DC) exhibited enhanced anti-corrosive properties compared to the system obtained under PC (Sn-Cu-Ni-PC) after reflowing. However, prior to reflowing, the opposite effect was observed, with Sn-Cu-Ni-PC showing improved anti-corrosive properties. These changes in anti-corrosive behavior were attributed to the modification of the alloy’s composition during the reflowing process.

## 1. Introduction

Tin and tin alloys possess excellent solderability; therefore, they are extensively used in the electronics industry to bond electronic components [1]. Predominantly, printed circuit board manufacturing, semiconductors and connectors have extensively employed Sn-Pb alloys [2]. However, due to the toxicity of Pb, the European Union (Directive 2002/95/EC) restricts the use of tin-lead alloys in electrical and electronic equipment [3,4]. Since then, a variety of tin-based alloys have been formulated as alternatives to lead-based alloys [3,5,6,7,8,9]. Among the array of lead-free alloy candidates, Sn-0.7Cu-0.05Ni solders are popular [10,11,12]. The alloy is economic as it does not contain expensive elements such as Ag or rare earth metals, it has a melting temperature of 227 °C and it shows good anti-corrosive and solderability properties [13,14,15].

The electrodeposition of solderable coatings is a low-cost technique that enables the production of deposits with any desirable thickness [7,16]. The majority of the electroplating processes available today are based on aqueous electrolytes [17,18,19,20,21]. However, sometimes they present significant drawbacks, including narrow potential windows and hydrogen evolution as a secondary reaction that could affect the cathodic efficiency and deposit characteristics. In addition, the aqueous electrolytes traditionally involved in industrial processes contain several hazardous chemical compounds [22]. Furthermore, the electrodeposition of some binary and ternary alloys proves challenging in conventional water-based solutions due to the high differences between the redox potentials of the metals to be co-deposited [23]. A promising alternative to overcome these limitations is based on the use of ionic liquids (ILs). Deep eutectic solvents (DESs) represent a class of ILs which are cost-effective, biodegradable, and non-toxic, and they can be easily synthetized by mixing certain hydrogen bond donors (HBDs) and acceptors (HBAs) in the right proportions. They have been successfully used as electrolytes during the electrodeposition of a variety of metals and alloys [22,24]. Moreover, ILs and DESs solvents have been utilized in a broad spectrum of electrochemical applications at the pilot plant level or commercial scale [25,26,27]. A spin-off company from the University of Leicester, Scionix Ltd. (London, UK), is one of the world’s largest manufacturers and distributors of ionic liquids for the plating of metals (Cr, Al, Co, Ni, Cu, Zn, Sn, Pb and Ag) and alloys (Cu/Zn, Zn/Co and Zn/Sn) for industrial applications [28].

The electrodeposition of the Sn-Cu-Ni alloy from choline chloride-based deep eutectic solvents has been previously investigated [29,30,31]. Sn-Cu-Ni alloy films were electrodeposited on Ni foam from a 1 choline chloride/2 ethylene glycol DES-based electrolyte, using nickel matte as the raw material, under potentiostatic conditions at various fixed potentials of −0.4, −0.6 and −0.8 V vs. Ag ref. [31]. It has been shown that the applied cathodic potential strongly influences the morphology of the deposits. In previous works, our group explored the synthesis of Sn-Cu-Ni alloy under direct current (DC) and pulsed current (PC) plating modes using choline chloride mixed with ethylene glycol at a 1:2 molar ratio [29,30]. The use of other types of DES-based electrolytes, such as the eutectic mixture of choline chloride/malonic acid (1:1 molar ratio), led to non-adherent deposits. The formulation of the electrolyte has been adjusted to attain the industrial stoichiometry of the lead-free soldering alloy, Sn-0.7Cu-0.05Ni. The closer formulation was obtained under DC electrodeposition: Sn99.29-Cu0.65-Ni0.06. For the pulsed plating electrodeposition, it fixed the value of the duty cycle at 16.7 and the current density at 8 mA/cm^2^, identical to the value applied during the electrodeposition under the DC mode (Sn-Cu-Ni-DC system), while the frequency varied from 16.67 Hz to 0.0167 Hz [30]. Among the different investigated pulsed plating parameters, electrodeposition was performed at a frequency of 1.67 Hz, corresponding to T_ON_ = 100 ms and T_OFF_ = 500 ms (ON and OFF represent time durations of the pulse), showing optimal results in term of current efficiency and grain size (Sn-Cu-Ni-PC system). The use of PC has been proved to improve the morphology of the deposit. The grain size decreased from 16.7 ± 4.1 µm for the Sn-Cu-Ni-DC system to 4.3 ± 1.7 µm for the Sn-Cu-Ni-PC one. Regarding the alloy’s composition, small variations are observed when changing the plating mode from DC to PC. Sn is the major constituent of the system, with contents of 99.29 ± 0.25 wt.% and 98.66 ± 0.81 wt.% for Sn-Cu-Ni-DC and Sn-Cu-Ni-PC, respectively. The concentration of copper decreases from 0.65 ± 0.10 wt.% to 0.44 ± 0.13 wt.% when the plating parameters are changed from DC to PC. Regarding nickel concentration, an increase was noticed for the selected PC plating parameters from 0.06 ± 0.02 wt.% to 0.90 ± 0.20 wt.%. Corrosion investigations conducted at both the macro- and micro-scales indicated that the utilization of the pulse current enhanced the corrosion resistance of the alloy. The potentiodynamic polarization investigations showed a lower corrosion current for the Sn-Cu-Ni-PC system, and electrochemical impedance spectroscopy (EIS) analysis revealed higher film resistance for the PC electrodeposited alloy.

Since the Sn-Cu-Ni alloys had applications as lead-free solders in the electronics industry, the present work investigated the influence of a reflow process (using an industrial oven from an electronic company) on the morphology, composition and anti-corrosive properties of the electrodeposited Sn-Cu-Ni alloy on a Cu substrate under the DC and PC plating modes.

## 2. Materials and Methods

### 2.1. Electrochemical Preparation of Sn-Cu-Ni Ternary Alloy and Reflowing Process

The used deep eutectic solvent, namely ILEG, was prepared by mixing choline chloride (ChCl) (Merck, >98%, Rahway, NJ, USA) with ethylene glycol (Silal Trading Bucharest, 99%, Bucharest, Romania) at a 1:2 molar ratio. The mixture was subjected to heating in the range of 80 to 90 °C, leading to the formation of a homogeneous, transparent liquid. Subsequently, 500 mM SnCl_2_·2H_2_O (SnCl_2_·2 H2O, Acros Organics, >97%, Geel, Belgium), 0.055 mM NiCl_2_·6H_2_O (NiCl_2_·6 H_2_O, Lach-Ner Ltd., 99%, Neratovice, Czechia) and 0.345 mM CuCl_2_·2H_2_O (CuCl_2_·2H_2_O, VWR Chemicals, 99%, Suwanee, GA, USA) matching the desired alloy composition were introduced into the electrolyte at 90 °C while maintaining continuous mild stirring and heating. The electrolyte composition was previously optimized [29]. The content of water in the electrolyte was found to be in the range of 0.1–0.5% using Karl–Fisher titration (TitroLine17500 KF titrator). The electrolyte was stored in sealed glass containers to minimize its exposure to air and atmospheric moisture.

A two-electrode setup was used for the electrodeposition of the ternary alloy. As an anode, a graphite plate with a surface area of 10 cm^2^ was used. As a cathode, copper sheets with a thickness of 0.2 mm and a working area of 7.5 cm^2^ were employed. Both electrodes were connected to a pulsed reversed power supply, specifically the pe86CB3HE model from Plating Electronic GmbH. Before the plating process, the copper sheets underwent a surface preparation, which involved (i) cleaning with acetone and (ii) chemical etching with a H_2_SO_4_:H_2_O_2_:H_2_O solution at a ratio of 5:10:85 vol.% for 30 s at room temperature. The specimens were subsequently washed with deionized water and left to air-dry. The Sn-Cu-Ni alloy was electrodeposited onto Cu substrate under either the direct current (DC) or pulsed current (PC) plating mode. The electrodeposition parameters are detailed in Table 1. The alloy was plated for 30 min at a temperature of 60 °C under stirring from the electrolyte mentioned above. Following the electrochemical procedure, the resulting alloys underwent a cleaning sequence, which involved rinsing with hot deionized water and then acetone. Finally, the coatings were dried using ambient air.

**Figure 1:**
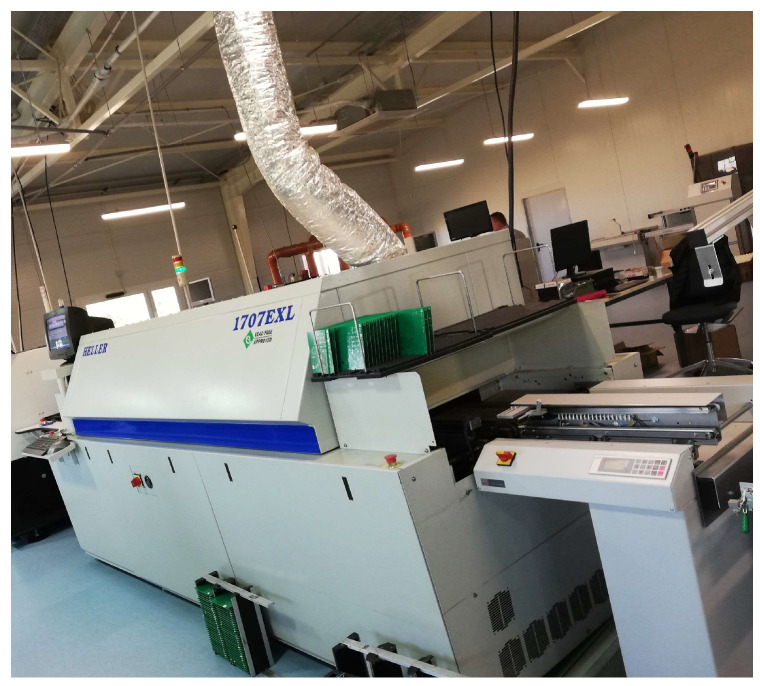
Industrial oven from MIBATRON S.R.L utilized for reflowing of Sn-Cu-Ni ternary alloy.

Following electrodeposition, both systems were exposed to a reflowing process using an industrial oven available at MIBATRON S.R.L (https://www.mibatron.ro/ (accessed on 5 December 2023)), a Romanian company specializing in automated PCB assembly. The samples were subjected to a gradual increase in the temperature from 160 °C to 260 °C for 7 min, ensuring the melting of the ternary alloys that have a melting point of 229.4 °C for the Sn-Cu-Ni-DC system and 229.6 °C for the Sn-Cu-Ni-PC system, as has been previously determined [30]. Figure 1 presents the industrial oven used.

### 2.2. Structural and Morphological Characterization

The morphological and elemental composition analyses of the alloys before and after the reflowing process were conducted with Scanning Electron Microscopy (SEM) from Hitachi SU 8230 (Tokyo, Japan) provided with Energy Dispersive X-ray spectroscopy (EDX) from Oxford Instrument (High Wycombe, UK). The crystallographic structure of the alloys was examined using X-ray diffraction (XRD), involving an X-ray diffractometer from Rigaku (Tokyo, Japan) (9 kW, CuKα, λ = 0.15406 nm) from 20 to 90°.

### 2.3. The Assessment of the Corrosion Behavior

The corrosion performance of Sn-Cu-Ni alloys synthetized via electrodeposition on a Cu substrate and subjected to a reflowing process was investigated through potentiodynamic polarization measurements and electrochemical impedance spectroscopy (EIS). For potentiodynamic polarization analysis, the sweep rate was maintained at 1 mV/s, while for EIS, the measurements were recorded as the open-circuit potential (OCP), the frequency range varied from 100 kHz to 100 mHz, and the 10 mV AC voltage was fixed. The recorded data were analyzed using ZView 2.4 software from Scribner Association Inc. (Southern Pines, NC, USA). The experiments were carried out in aerated 0.5 M NaCl solution, involving a PARSTAT 4000 potentiostat operating in VersaStudio software (Version 2.1). The experimental setup included a working electrode (WE) with a defined surface area of 0.63 cm^2^, a Pt plate serving as the counter electrode (CE), and an Ag/AgCl reference electrode (RE).

In addition, the alloys were exposed in 0.1 M NaCl for a period of 168 h at room temperature. The specimens were investigated periodically after 24, 72, 96 and 168 h of immersion using SEM-EDX.

## 3. Results and Discussion

**Figure 2:**
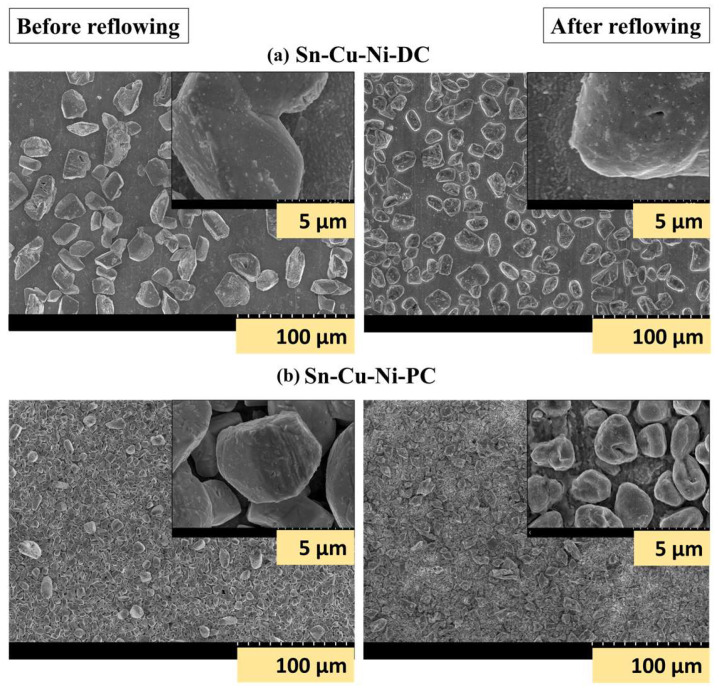
SEM micrographs at different magnifications of (**a**) Sn-Cu-Ni-DC and (**b**) Sn-Cu-Ni-PC specimens before and after reflowing process.

Previous studies have demonstrated that the Sn-Cu-Ni alloy can be successfully electrodeposited on Cu substrate under the DC and PC plating modes from choline chloride-based deep eutectic solvents [29,30]. In both cases, the current density was set at 8 mA/cm^2^. A frequency of 1.67 Hz was found to be optimal for the alloy electrodeposition under the PC mode. The use of PC facilitates the refinement of the grain size of the alloy, as illustrated in Figure 2. To assess the impact of the reflowing process on Sn-Cu-Ni alloy coatings, copper sheets (with a surface area of 7.5 cm^2^) coated with the alloy under both the direct current (Sn-Cu-Ni-DC) and pulsed current (Sn-Cu-Ni-PC) modes were subjected to the reflow operation in an industrial oven. The temperature in the industrial oven was progressively raised from 160 °C to 260 °C over a 7-min interval. The melting point of both systems was previously determined to be 229.4 °C for the Sn-Cu-Ni-DC system and 229.6 °C for Sn-Cu-Ni-PC system [30]. Therefore, the melting of the alloy occurred during the reflowing process setup.

Figure 2 presents the SEM micrographs of the Sn-Cu-Ni-DC and Sn-Cu-Ni-PC systems before and after exposure to reflowing. As illustrated in Figure 2, after reflowing, the grains are partially melted, as the SEM micrographs show. For the alloy prepared under PC conditions, the grains were interconnected with a metallic layer formed as a result of the grains melting.

**Figure 3:**
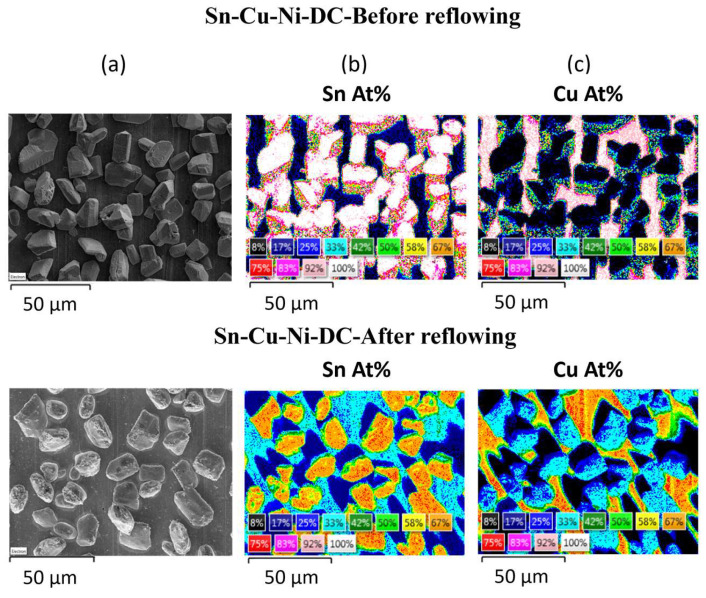
Sn-Cu-Ni-DC alloy before and after reflowing process: (**a**) SEM micrograph; EDX quantitative maps for (**b**) Sn and (**c**) Cu.

**Figure 4:**
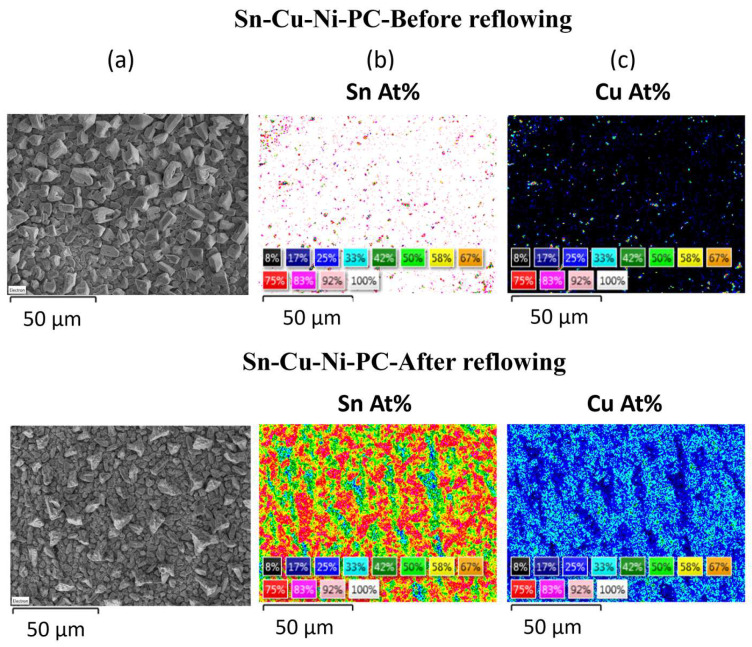
Sn-Cu-Ni-PC alloy before and after reflowing process: (**a**) SEM micrograph; EDX qualitative maps for (**b**) Sn and (**c**) Cu.

In addition, subjecting the alloys to the reflowing treatment resulted in a modification of their composition. An EDX quantitative analysis was performed before and after reflowing by recording the elemental maps of the main components, as shown in Figure 3 and Figure 4. The variation in the atomic concentrations of Sn or Cu between 8 (black) and 100 at.% (white) is represented in these maps with different colors.

Prior to reflowing, the grains of both systems predominantly consisted of Sn, as it is the primary constituent of the alloy. The concentration of Sn within the grains varied between 92 and 100 at.% for both samples. Sn-Cu-Ni-DC system exhibits a lower concentration of Sn between the grains ranging from 8 to 17 at.%. In contrast, for the Sn-Cu-Ni-PC system, the copper substrate is nearly entirely coated by grains, with minimal gaps between the grains exposing the substrate.

After reflowing, it is observed in both systems that the grains exhibit an increased Cu content due to its diffusion from the substrate into the grains. For the Sn-Cu-Ni-DC sample, the Sn content within the grains diminishes towards 67–75 at%, while the inter-grain spaces experience an enrichment in tin content attributed to the partial melting of the grains, leading to a Sn content of 25–33 at.%. In the case of Sn-Cu-Ni-PC system, the concentration of Sn varies between 25 and 75 at.%. Following the reflow process, the uniform distribution of Sn observed prior to reflowing has undergone a significant transformation. Post-reflow, distinct regions exhibit notable variations in tin concentration, resulting in areas characterized by both higher (75 at.%) and markedly lower Sn concentrations (17–25 at.%). This observed non-uniformity in Sn distribution highlights the impact of the reflow process on the elemental arrangement.

**Figure 5:**
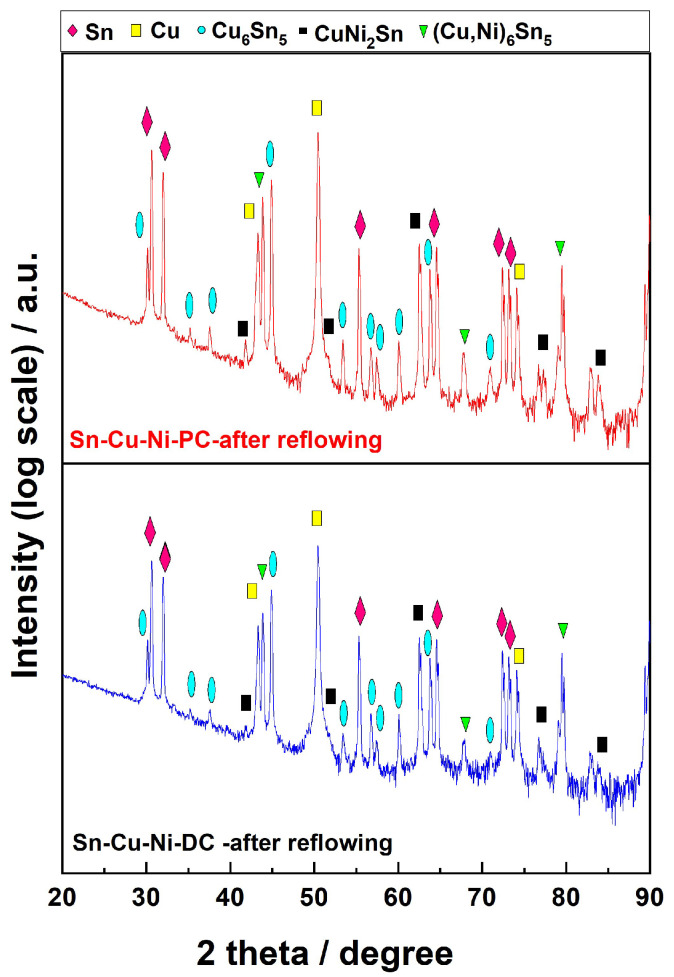
X-ray diffraction pattern of SnCuNi-DC and SnCuNi-PC coatings on copper sheets after reflowing process.

The variations in the alloy’s composition detected through EDX analysis have also been correlated with X-ray diffraction. Figure 5 illustrates the XRD pattern of the Sn-Cu-Ni alloy electrodeposited under DC and PC after exposure to reflowing process. For a better analysis, the vertical axis has been plotted in logarithmic scale. As previously reported, the peaks associated with the Cu substrate (card No. 00-004-0836) and tetragonal Sn (card No. 00-004-0673) have been identified, as well as the ones corresponding to monoclinic Cu_6_Sn_5_, (card No.: 01-076-2703), hexagonal (Cu, Ni)_6_Sn_5_ (PCD Crystal Data #1905144) and cubic CuNi_2_Sn intermetallic (ICSD#103068) [31,32,33]. Following the reflow process, the XRD analysis highlighted the increased formation of Cu_6_Sn_5_, (Cu, Ni)_6_Sn_5_ and CuNi_2_Sn intermetallic phases. Even when comparing the two systems, the sample obtained under PC exhibits higher intensity in the peaks associated with intermetallic phases.

**Figure 6:**
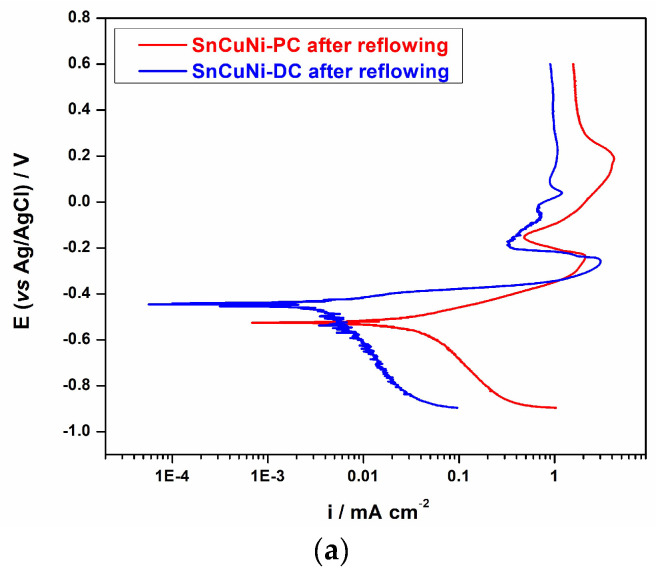

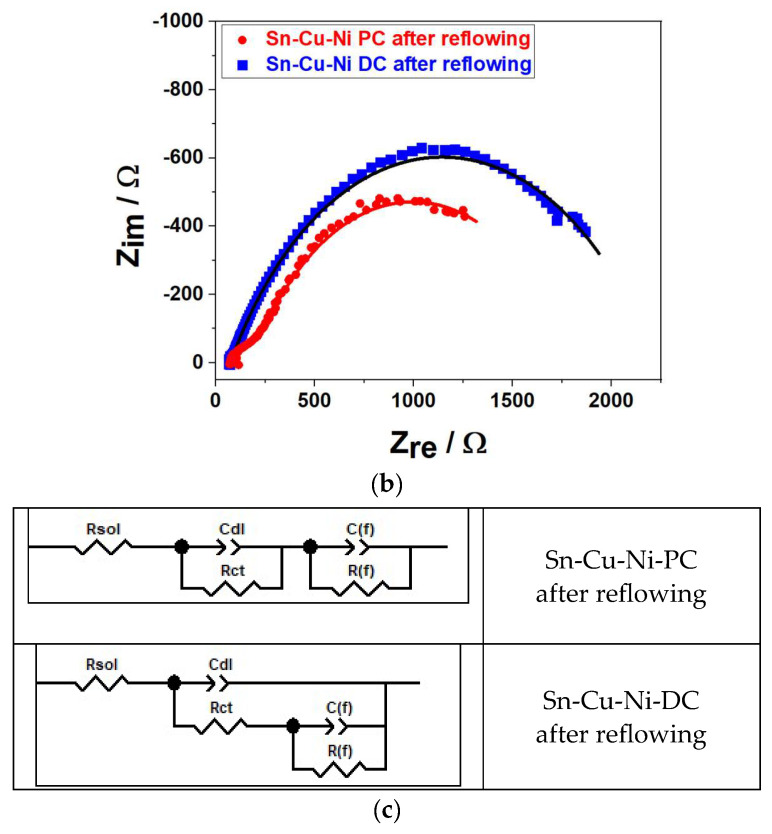
(**a**) Potentiodynamic polarization curves in semilogarithmic coordinates (25 °C, 1 mV/s), (**b**) Nyquist plots at the open-circuit potential, in 0.5 M NaCl, for the electrodeposited Sn-Cu-Ni-DC and Sn-Cu-Ni-PC alloys on Cu after the reflowing process and (**c**) the electrical equivalent circuits used to fit the impedance spectra.

Regarding the corrosion protection, the performance of Sn-Cu-Ni alloy coatings was evaluated by examining the recorded potentiodynamic polarization curves and electrochemical impedance spectra (EIS) in a 0.5 M NaCl solution. A typical polarization graph in semilogarithimic coordinated for the electrodeposited Sn-Cu-Ni alloys under both the DC and PC plating modes following their exposure to the reflowing process is shown in Figure 6. By extrapolating from the Tagel plots, the corrosion potential (E_corr_) and corrosion current density (i_corr_) were found. It has previously been shown [30] that the as-prepared Sn-Cu-Ni-PC specimens exhibited better anti-corrosive properties compared to the Sn-Cu-Ni-DC ones. Even the corrosion potential was quite similar, E_corr_ ~−0.60 V vs. Ag/AgCl, and the corrosion current of Sn-Cu-Ni-PC alloy was much lower (2.7 µA/cm^2^) than the one of the samples prepared under the direct current mode (9.6 µA/cm^2^) [30]. Nevertheless, with reflowing, the systems’ anti-corrosive properties were altered. The corrosion potential of Sn-Cu-Ni-DC alloy shifted to more positive values, E_corr_ = −0.45 V vs. Ag/AgCl, and the current density, 2.76 µA/cm^2^, was one order of magnitude lower than the one determined for the PC system, namely E_corr_ = −0.53 V vs. Ag/AgCl and i_corr_ = 21.1 µA/cm^2^, respectively.

When scanning in the anodic direction, the polarization curve exhibits three distinct potential ranges, highlighting key corrosion characteristics. An increase in the current density is observed in the first region upon scanning from the E_corr_ in the anodic direction, which is ascribed to the active dissolution of the Sn-Cu-Ni alloy. The alloy grains, which are primary constituents of tin, are in contact with the copper substrate. The dissolution reaction typically occurs for the metals with a lower standard electrode potential [34]. Considering the more negative standard electrode potential of the Sn/Sn^2+^ compared to Cu/Cu^2+^, tin functions as the anode in the galvanic cell, leading to its preferential corrosion. The active dissolution of the alloy continues until the concentration of the dissolved metals reaches the passivation potential (*E_p_*), and a value of ~−0.25 V is measured for both samples. Upon further scanning, a passive film begins to form. As tin ions move through the aqueous solution, a metal oxide precipitate forms on the surface of the sample. The passivation current density presents a higher value for the sample prepared in DC (3.018 mA/cm^2^) compared to the sample obtained in PC (2.16 mA/cm^2^). In both cases, in the passive region, the current decreases by one order of magnitude, with values of 0.32 mA/cm^2^ and 0.47 mA/cm^2^ recorded for Sn-Cu-Ni-DC and Sn-Cu-Ni-PC, respectively. Compared to the sample obtained under PC, three different passive films may appear for the sample prepared under direct current conditions, as evidenced by the subsequent increase in current density up to 0.67 mA/cm^2^ following the initial passive region, its breakdown and another film formation at 0.91 mA/cm^2^. Finally, the third potential region, after +0.2 V, corresponds to the breakdown of the passive films as a result of the presence of chloride ions in the medium. The description of the corrosion aligns well with the findings documented in the literature [15,34,35].

Figure 6b shows the recorded EIS spectra as Nyquist diagrams, where the diameters of the semicircles give an indication of the polarization resistance Rp, as a measure of the corrosion performance. It is clear that a larger diameter for the sample prepared under DC indicates a lower corrosion current than that corresponding to the sample prepared under PC, confirming the data from the polarization curves. In order to fit the experimental electrochemical impedance data and find more quantitative information on the corrosion performance of the investigated specimens, the electrical equivalent circuits presented in Figure 6c have been proposed and tested. Those showing the lowest chi-squared and minimum error values have been selected. During the fitting of the experimental data, constant phase elements (CPEs) instead of capacitances were used in order to more accurately model the non-ideal capacitive behavior of the electrode/electrolyte interface [36]. Rsol represents the ohmic resistance of the solution. Cdl and Rct represent the double layer capacitance and charge transfer resistance, while C(f) and R(f) represent the capacitance and resistance of the layer, associated with the oxide layer/alloy interface. The EIS parameters for the proposed electrical equivalent circuit are showed in Table 2.

The change in the anti-corrosive properties could be associated with the modification of the alloy composition determined via EDX and X-ray analysis. The increased formation of intermetallic phases as a result of exposure to reflow process influenced the protective properties of the ternary alloy. The intermetallic phases act as cathodes in the galvanic cell, promoting the dissolution of Sn [37,38].

**Figure 7:**
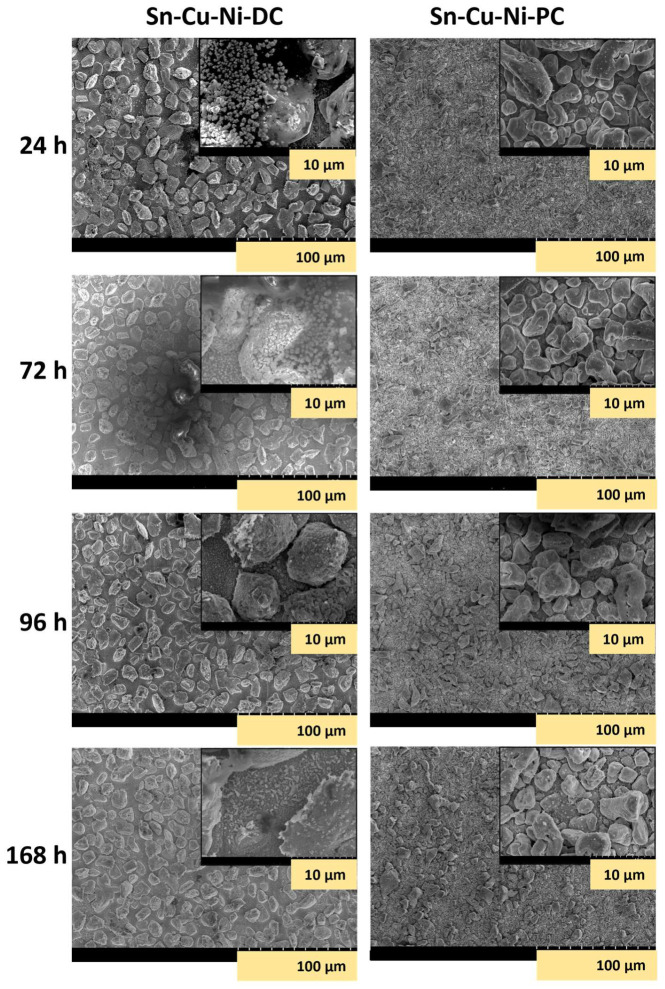
SEM micrographs of Sn-Cu-Ni-DC and Sn-Cu-Ni-PC alloy specimens subjected to the reflowing process after 24 h, 72 h, 96 h and 168 h of immersion in a 0.1 M NaCl solution.

**Figure 8:**
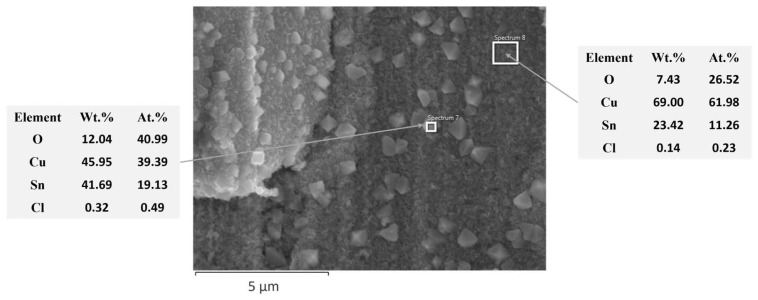
EDX quantitative analysis on Sn-Cu-Ni-DC specimen subjected to the reflowing process after 168 h of immersion in 0.1 M NaCl solution (Spectra 7 and 8 represent the regions analyzed).

**Figure 9:**
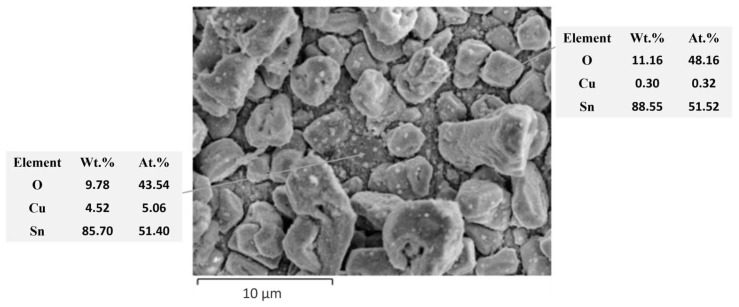
EDX quantitative analysis of the Sn-Cu-Ni-PC specimen subjected to the reflowing process after 168 h of immersion in a 0.1 M NaCl solution.

In another sequence of experiments, the Sn-Cu-Ni-DC and Sn-Cu-Ni-PC specimens subjected to the reflowing process were immersed in 0.1 M NaCl solution for 168 h, with periodical examinations after 24 h, 72 h and 96 h to assess the morphology and composition modifications during exposure, as illustrated in Figure 7, Figure 8 and Figure 9.

Figure 7 presents the recorded SEM micrographs of both systems at intervals of 24 h, 72 h, 96 h and 168 h during exposure to the corrosive environment.

After 24 h of immersion, the Sn-Cu-Ni-DC specimen showed the development of corrosion products as crystal and spongy plates on its surface. In contrast, on the Sn-Cu-Ni-PC surface, no corrosion products were identified. Wang et al., previously reported the formation of corrosion products with a crystal shape, composed of SnO_2_/SnO, for SAC305 solder joints [39,40]. Prior to reflowing, the opposite effect was observed. The Sn-Cu-Ni-PC sample presented corrosion products in the form of crystal shape and spongy plates after 24 h of immersion, while the Sn-Cu-Ni-DC did not exhibit any corrosion products. Therefore, the modification of the alloy’s composition as a result of the exposure to the reflow process influences its protective characteristics, as has been previously noted through the potentiodynamic polarization curves and EIS analysis. After reflowing, in the case of Sn-Cu-Ni-DC specimen, the space between the grains showed a higher Sn content, due to the partial melting of the grains. As a result, the Cu substrate was shielded against aggressive chloride ions with a protective layer of Sn. That layer, upon exposure to an aggressive environment, could then form a protective passive film.

After 72 h of immersion, no signs of corrosion products were observed on the surface of Sn-Cu-Ni-PC system. In contrast, the Sn-Cu-Ni-DC specimen exhibited increased formation of corrosion products. In fact, on the surface of the Sn-Cu-Ni-PC specimen, no corrosion products were detected up to 96 h of immersion. After 168 h of exposure, both systems showed a partial dissolution of the alloy grains. EDX investigations, as illustrated in Figure 8 and Figure 9, revealed the presence of Sn, O, Cu and Cl elements, suggesting the formation of tin oxides and chlorides as corrosion products. Tin, as the primary component of the alloy, acted as an anode in the galvanic couple with Cu from the substrate, leading to its preferential corrosion and resulting in Sn-based corrosion products. Since the spaces between the grains for Sn-Cu-Ni-DC systems are filled with a protective layer of Sn, upon exposure to an aggressive environment, they will form a protective passive film with a spongy plate morphology. Corrosion products with crystal-like morphologies were ascribed to SnO_2_/SnO. Prior to reflowing, when the Sn-Cu-Ni-PC system was exposed to 168 h of immersion in 0.1 M NaCl, corrosion products as crystals and spongy plates appeared after the first 24 h of exposure. Subsequently, after 168 h of conditioning, the initially formed film became more compact and covered the surface of the deposit, acting as a barrier against aggressive chloride ions [30]. Upon exposure to the reflowing process, the formation of a compact film on the alloy was no longer evident when the system was immersed in a 0.1 M NaCl solution. Therefore, there was a transformation in the morphology of the corrosion products, which could be associated with the modification of the anti-corrosive properties of the Sn-Cu-Ni alloys after exposure to the reflowing process.

## 4. Conclusions

Sn-Cu-Ni lead-free solder alloys were electrodeposited on copper substrate under the DC and PC plating modes and subjected to a reflowing process using an industrial oven. After exposure to reflowing process, the following may be concluded:

- A change in the alloy’s composition occurred. Prior to reflowing, the grains consisted mainly of Sn for both systems. The Sn-Cu-Ni-PC specimen exhibited narrow gaps among its grains, whereas the Sn-Cu-Ni-DC one displayed larger grain morphology with an exposed Cu substrate spaces. Following reflow, Cu from the substrate diffused into the grains, and Sn from the grains diffused both between the grains and onto the substrate. The Sn-Cu-Ni-PC system has become non-uniform in composition, with certain regions showing elevated concentrations of tin, while others show markedly lower tin concentrations.
- The XRD analysis showed the formation of more intermetallic after reflowing. Comparing both systems, the sample obtained under PC exhibits higher intensity in the peaks associated with intermetallic phases.
- The corrosion performance after reflowing was investigated via potentiodynamic polarization and EIS spectroscopy in a 0.5 M NaCl solution. Prior to reflowing, the Sn-Cu-Ni-PC system exhibited improved anti-corrosive properties with lower corrosion current density. However, after reflowing, the opposite effect was observed, as Sn-Cu-Ni-DC presented more electropositive corrosion potential and lower current density. The changes in the anti-corrosive properties could be associated with the modification of the alloy’s composition. The SEM analysis during continuous immersion in 0.1 M NaCl solution for 168 h revealed that the Sn-Cu-Ni-DC system formed Sn-based corrosion products with crystal shapes and spongy plates after 24 h of immersion, while the Sn-Cu-Ni-PC system presented corrosion products on its surface only after 96 h of immersion. Since Sn diffuses from the grains, in the DC system, the space between the grains is covered with Sn. Upon exposure to the aggressive environment, in that region, we observed the formation of a passive film with a spongy plate morphology that protects the substrate from the chloride ions.

## Figures and Tables

**Table 1:** Electroplating parameters for Sn-Cu-Ni alloy synthesis (Reprinted from Surface and Coatings Technology, Vol 477, State S. et al., Electrodeposited Sn-Cu-Ni alloys as lead-free solders on copper substrate using deep eutectic solvents: The influence of electrodeposition mode on the morphology, composition and corrosion behaviour, 130324, Copyright (2024), with permission from Elsevier) [30].

System Type	On- and Off-Time Duration of the Pulse	Frequency (Hz)	Duty Cycle (%)	Current Density (mA/cm^2^)
Sn-Cu-Ni-DC	-	-	-	8
Sn-Cu-Ni-PC	Ton = 100 ms Toff = 500 ms	1.67	16.7	8

**Table 2:** Fitting results of impedance spectra for the Sn-Cu-Ni ternary alloys after reflowing using the equivalent circuits proposed in Figure 6c.

Parameter	System	
	Sn-Cu-Ni-DC after Reflowing	Sn-Cu-Ni-PC after Reflowing
R_sol_/Ω cm^2^	46.5	44.8
R_CT_/Ω cm^2^	124.9	87.2
C_dl (CPE 1)_/µF cm^−2^	195.8	201.9
n_(CPE 1)_	0.65	0.58
R_F_/Ω cm^2^	1213.4	1011.2
C_F (CPE 2)/_µF cm^−2^	18.1	715.8
n_(CPE 2)_	0.77	0.69
χ^2^	4.32 × 10^−3^	4.91 × 10^−3^

## Data Availability

Data are contained within the article.
